# Protocol for Callus Induction and Somatic Embryogenesis in Moso Bamboo

**DOI:** 10.1371/journal.pone.0081954

**Published:** 2013-12-11

**Authors:** Jin-Ling Yuan, Jin-Jun Yue, Xiao-Li Wu, Xiao-Ping Gu

**Affiliations:** Research Institute of Subtropical Forestry, Chinese Academy of Forestry, Fuyang, Zhejiang, P. R. China; United States Department of Agriculture, United States of America

## Abstract

Moso bamboo [*Phyllostachys heterocycla* var. pubescens (Mazel ex J. Houz.) Ohwi] is one of the most important forest crops in China and the rest of Asia. Although many sympodial bamboo tissue culture protocols have been established, there is no protocol available for plantlet regeneration as indicated by callus induction for monopodial bamboos, such as Moso bamboo. In the present report, embryogenic callus induction, embryoid development, and germination were established for Moso bamboo from zygotic seed embryos. Callus was initiated from zygotic embryos after 10–20 d culture on MS media supplemented with 4.0 mg/L 2, 4-D and 0.1 mg/L zeatin (ZT). About 50% of the explants produced calli, and nearly 15% of the calli were found to be embryogenic in nature. These embryogenic calli can be subcultured for proliferation in the Murashige and Skoog media (MS) supplemented with 0.5–2.0 mg/L 2, 4-D. These calli were found to have maintained their capacity for regeneration even after one year of subculture. The viable somatic embryoids regenerated in medium containing 5.0–7.0 mg/L ZT. Nearly 5% of the calli were found capable of regenerating into plantlets directly in MS medium containing 5.0–7.0 mg/L ZT. Root growth was more pronounced when the plantlets were transferred to medium containing 2.0 mg/L NAA. After 30 days of subculture, the plantlets were transferred to a greenhouse.

## Introduction

Bamboo is a fast-growing plant, and the material it yields can be used for many purposes. As such, bamboo is an important resource in Asian agricultural, fishery, construction, paper, plywood, and artisanal industries [1], [2]. However, conventional bamboo breeding is limited by the genera's long and irregular flowering, poor seed setting, low seed viability, and unpredictable seed production [3]–[6]. Modern biological techniques, such as gene transformation and protoplast fusion may help solve these problems.

Plant transformation via agrobacterium-mediation or gene gun involves somatic embryogenesis to produce plant material [7], [8]. Based on the morphological differences in bamboo rhizomes, bamboo species can be divided into two clades, sympodial and monopodial [9]. At present, somatic embryogenesis protocols have been established for a number of sympodial bamboo species [10]–[18]. The monopodial rhizome structure of the running bamboos is long and the rhizomes spread quickly, making it a top choice for reforestation and industrial cultivation. There are fewer successful and convenient plantlet regeneration systems for monopodial bamboo [19]–[22]. It is necessary to establish simple and cost-effective plantlet regeneration in monopodial bamboos.

Moso bamboo [*Phyllostachys heterocycla* var. *pubescens* (Mazel ex J. Houz.) Ohwi), a native perennial with monopodial growth habits, is the most economically and ecologically valuable bamboo species in China [23], [24]. Almost every part of Moso bamboo is usable: young shoots and the tender shoot cover are edible, culms are used as raw material in architecture and can be processed into plywood and floorboards, rhizomes are used in handicrafts, and branches are made into brooms [23]. Moso bamboo has a wide distribution, from subtropical to semi-tropical areas throughout the world, where it has naturalized. Specifically, it was introduced to Japan in 1736, to Europe in 1880 and to the United States in about 1890 [24]. In China alone it can be found in an area of nearly 30,000 hm^2^.

The present work reports a reliable callus induction and somatic embryogenesis protocol for monopodial Moso bamboo. The embryoids were induced from zygotic embryos of Moso bamboo in medium containing 2, 4-D. The embryoids can be formed 20–30 days after subcultured in medium containing zeatin. The embryoids germinate after subsequent incubation in medium containing the cytokinin. Improved root growth and development occurred when the plantlets were transferred to medium containing NAA. This protocol may serve as the first step in the establishment of gene-transformation technology suitable for use in the bamboo industry.

## Materials and Methods

### Explants and sterilization

**Figure 1:**
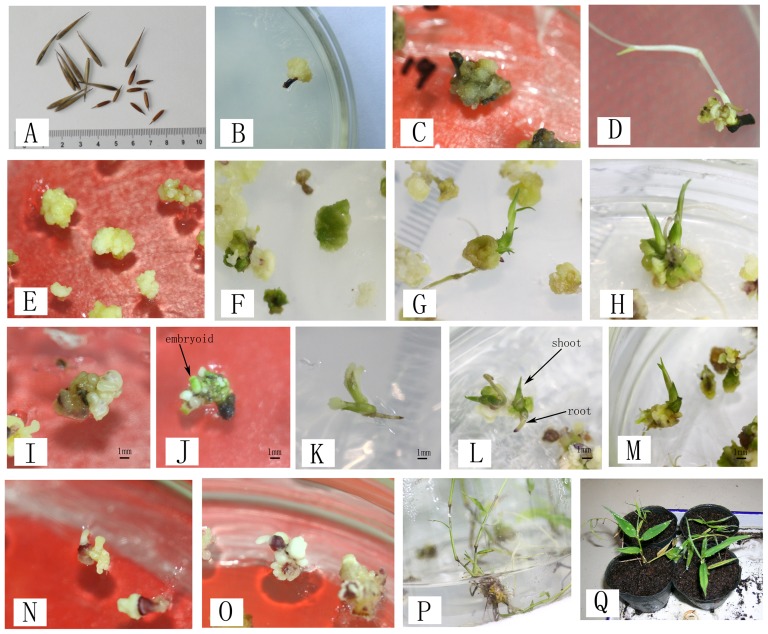
Callus induction, embryogenic callus initiation, and plantlet regeneration in Moso bamboo and levels used to produce these images of each compound. A. Explants (seeds) before incubation. B. Embryogenic calli induced on MS medium containing 4 mg/L 2, 4-D. C. Vitrified calli that did not subsequently proliferate or regenerate in NB induction medium. D. Callus formation accompanied with the zygotic germination of the explants (seeds) at 1 mg/L 2, 4-D. E. Subculture of light yellow embryogenic calli on MS supplemented with 2 mg/L 2, 4-D. F. Calli turning green after 7 days of incubation on medium containing 0.1 mg/L thidiazuron (TDZ). G. Shoot and root regeneration in 2.0 mg/L zeatin medium. H. Shoot and root regeneration in 5.0 mg/L zeatin medium. I. Spherical embryoids formed in 5.0 mg/L zeatin medium. J. Greening of spherical embryoids under light in 5.0 mg/L zeatin medium. K. Elongation and development of embryoids in 5.0 mg/L zeatin medium. L. Germination of embryoids in 5.0 mg/L zeatin medium. M. Shoots and roots of embryoids in 5.0 mg/L zeatin medium. N. Light yellow, transparent embryoids that turned purple at the area connecting the radicle to the germ in 7.0 mg/L zeatin medium. O. White nontransparent embryoids that turned purple at the area connecting the radicle to the germ in 7.0 mg/L zeatin medium. P. Plantlet subcultured in MS medium supplemented with 2 mg/L NAA and 0.2 mg/L ZT. Q. Survival plantlet transferred to the greenhouse.

The samples were collected form an automatic flowering bamboo forest lies 25°12′ 36″ east longitude, 110°46′ 12″ north latitude in Guangxi Zhuang Autonomous Region, China, in Aug. 2010 (Figure 1A). This area was owned by a local landowner, who gave the permission for the collection of material for the present study. Seed explants were immersed in 75% (v/v) ethanol for 1 min and washed 4–5 times with sterile, distilled water. They were then treated with 2% sodium hypochlorite solution containing 0.1% Tween-80 for 15–20 min and rinsed 6–8 times in sterile, distilled water. They were then inoculated into individual 9 cm diameter petri dish containing 20 ml induction medium.

### Moso bamboo callus induction

Seed embryos of Moso bamboo were inoculated on different media for callus induction.

Single factor design was applied in the experiments. Firstly, five basal media (MS, NB, N6, B5, CC) (Murashige and Skoog, 1962; Wu et al. 1987; Chu, 1978; Gamborg et al., 1968; Potrykus et al., 1979), were compared in the effect of callus induction, with each containing 4 mg/L 2, 4-dichlorophenoxyacetic acid (2, 4-D). Then, four kinds of plant growth regulators, 2, 4-D at 4 levels (1, 2, 4, and 6 mg/L); kinetin (KT), benzylaminopurine (BA), and zeatin (ZT), each at 4 levels (0.1, 0.5, 1.0, and 3.0 mg/L), were evaluated for callus induction, MS medium was supplemented in each experiment.

500 mg/L proline (Pro), 500 mg/L glutamine (Gln), 300 mg/L casein hydrolysate (CH), 30 g/L sucrose, and 8.0 g/L agar were added in each experiment. The pH was adjusted to 5.8 before autoclaving (20 min, 121°C, 1.03 kg/cm^2^). Hormones were filter-sterilized and added after the media had cooled.

Each treatment included 3 replicates. Each replicate included 30 dishes with 7 seeds per dish. The embryos were incubated at 26°C in the dark. After 30–40 days of incubation, the ratios of callus, embryogenic callus, callus induction with zygotic embryo germination, zygotic embryo germination alone, and no reaction of explants were recorded.

### Callus proliferation and embryogenic calli regeneration

Yellow and pale white embryogenic calli derived from the seed embryos were incubated in solid MS medium plus 0.5–2.0 mg/L 2, 4-D and transferred every two weeks. After 4 months of proliferation, the calli were used for the regeneration test.

Embryogenic calli were incubated on different media for somatic embryogenesis and embryoid germination in the MS-based media. Single factor design was applied in the experiments. Primarily, four cytokinins, specifically KT and BA, each at 4 levels (1.0, 2.0, 3.0, and 5.0 mg/L); thidiazuron (TDZ) at 4 levels (0.05, 0.1, 0.3, and 0.5 mg/L); and ZT at 5 levels (1.0, 2.0, 3.0, 5.0, and 7.0 mg/L), were evaluated in calli regeneration. Then, 5 carbon sources (sucrose, glucose, maltose, levulose, or lactose) each at 30 g/L, and 4 metals (CuSO_4_, AgNO_3_, CoCl_2_, or H_4_SiO_3_) each at 5.0 mg/L, were also added in the medium for stimulating somatic embryogenesis and embryoid germination.

Each treatment included 3 replicates, each replicate included 5 dishes with 30 calli per dish. These calli were cultured at 26°C under a 12 h photoperiod with light supplied at an intensity of 50 µmol m^−2^s^−1^. After 3–5 days, the growth of the calli was recorded with respect to greening, shoot regeneration, and embryoid formation and germination every 2 days.

### Plantlet hardening and survival

Plantlets were transferred to MS medium supplemented with 10 g/L agar and 30 g/L sucrose treated with 2 mg/L naphthaleneacetic acid (NAA) and 0.2–0.5 mg/L ZT for one month. The hardening plantlets were then transferred to soil in the greenhouse.

### Statistical analysis

The data were summarized as mean ± standard deviation. One-way ANOVA, regression analysis was performed after arc sine transformation because they were percentage data. If the ANOVA indicated significant results, a Duncan's mean separation test was then performed [25]. All data were analyzed using SPSS ver. 13.0.

## Results and Discussion

### Moso bamboo callus induction

#### Effect of basal medium on callus induction

Callus was found to be induced from Moso bamboo seed embryos on all five basic media (MS, NB, N6, B5, CC) (Table 1). However, there were differences among the plants grown in the 5 different media with respect to callus initiation time, the relative number of embryogenic calli, and the relative number of calli induced with a zygotic embryo germination stage.

**Table 1:** Basal medium and callus induction in Moso bamboo seeds*.

Basal medium	Effect of callus induction				
	Callus induction (%)	Embryogenic callus (%)	Callus induction with zygotic embryo germination (%)	Zygotic embryo germination alone (%)	No reaction (%)
NB	36.32±1.47B	3.94±0.73AB	20.13±0.95BC	3.31±1.64	60.37±1.38A
N6	37.91±2.16B	2.98±0.70BC	25.58±1.92AB	6.53±0.79	55.56±2.27AB
B5	37.25±2.80B	3.93±0.35AB	27.42±2.46A	4.27±1.64	58.49±2.86A
CC	39.59±4.60B	1.56±0.10C	16.05±0.55C	5.45±3.97	54.96±6.42AB
MS	50.34±2.13A	5.97±1.82A	22.42±3.55ABC	4.01±1.79	45.65±3.67B

4mg/L 2, 4-D were added in each experiment. Values are the mean ± standard deviation. Values followed by different capital letters showed significant differences at the 0.01 probability level.

On MS, callus in 1–2 mm diameter was observed 7–10 d and became 3–4 mm in diameter 30 d (Figure 1B) after inoculation, and the MS media yielded the highest relative numbers of both callus (about 50%) and embryogenic callus (nearly 6%). Calli were observed in NB, N6, B5, and CC only after 12–20 d and with lower ratios of embryogenic callus induction (1.56–3.94%), most of the calli grew slowly and turned brown easily (Figure 1C). The results of a mean separation analysis showed MS medium to be the best for embryogenic callus induction (Table 1). In this way, MS basal medium was considered suitable for use as induction medium. It was used as the basal media for the rest of the study.

Moreover, 16.05%–27.42% explants were observed callus induction along with seed embryo germination (Figure 1D); 3.31%–6.53% explants germinated directly without callus induction; and 45.65%–60.37% explants had no reaction after inoculation, that is, the explants neither germination nor induced callus.

#### Effect of plant growth regulators on callus induction

**Figure 2:**
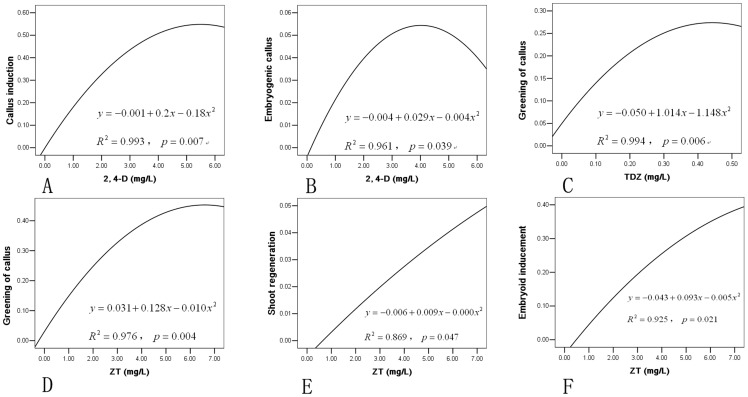
Regression analysis of callus induction and embryonic calli regeneration over the range of hormones from zygotic embryos of Moso bamboo*. A. Effect of 2, 4-D on callus induction. B. Effect of 2, 4-D on embyogenic callus formation. C. Effect of TDZ on greening of callus. D. Effect of ZT on greening of callus. E. Effect of ZT on shoot regeneration. F. Effect of ZT on embryoid inducement. * Values in the ordinate were data after arcsine transformation because the record were percentage.

Induction of callus occurred when the medium contained 2, 4-D, and the concentration of 2, 4-D affected callus induction. At a lower concentration of 2, 4-D (1 mg/L), zygotic germination of the explants (seeds) was higher and callus induction was lower and often accompanied the emergence of germination. While the ratio and rate of callus induction was increased with concentration of 2, 4-D. The association between the density of 2, 4-D and the effect of callus induction (namely, the rate of callus induction, and embryogenic callus) was evaluated using regression analysis, the results showed that the formation of callus and embryogenic callus over the range of 2, 4-D could be demonstrated by a quadratic regression equation, respectively (Figure 2A and 2B). In order to induce more embryogenic callus, a 2, 4-D concentration of 4 mg/L should be used.

Preliminary development indicates that a regeneration system for running bamboos such as Moso would be characterized by callus with slow proliferation, severe browning, and an inability to regenerate plantlets. For example, Han et al. and Chou et al. induced calli from Moso bamboo shoots, but they found those calli to be susceptible to severe browning, which hindered differentiation and formation of subcultures [26], [27]. Similarly, calli induced from Moso bamboo seeds exhibited slow growth, a light yellow color, loose texture, and severe vitrification [28]. In a *Bambusa edulis* study, cytokinin was found to be essential for embryogenic callus induction and maintenance [5]. The effects of cytokinins on Moso bamboo somatic embryogenesis were studied in this way. Germination of the explant embryos increased in the medium supplemented with cytokinins. However, KT and BA inhibited the induction of callus and completely blocked the induction of callus when the concentrations were higher than 0.5 mg/L. In this way, BA and KT were found to be unsuitable supplements for embryogenic callus induction in Moso bamboo.

**Figure 3:**
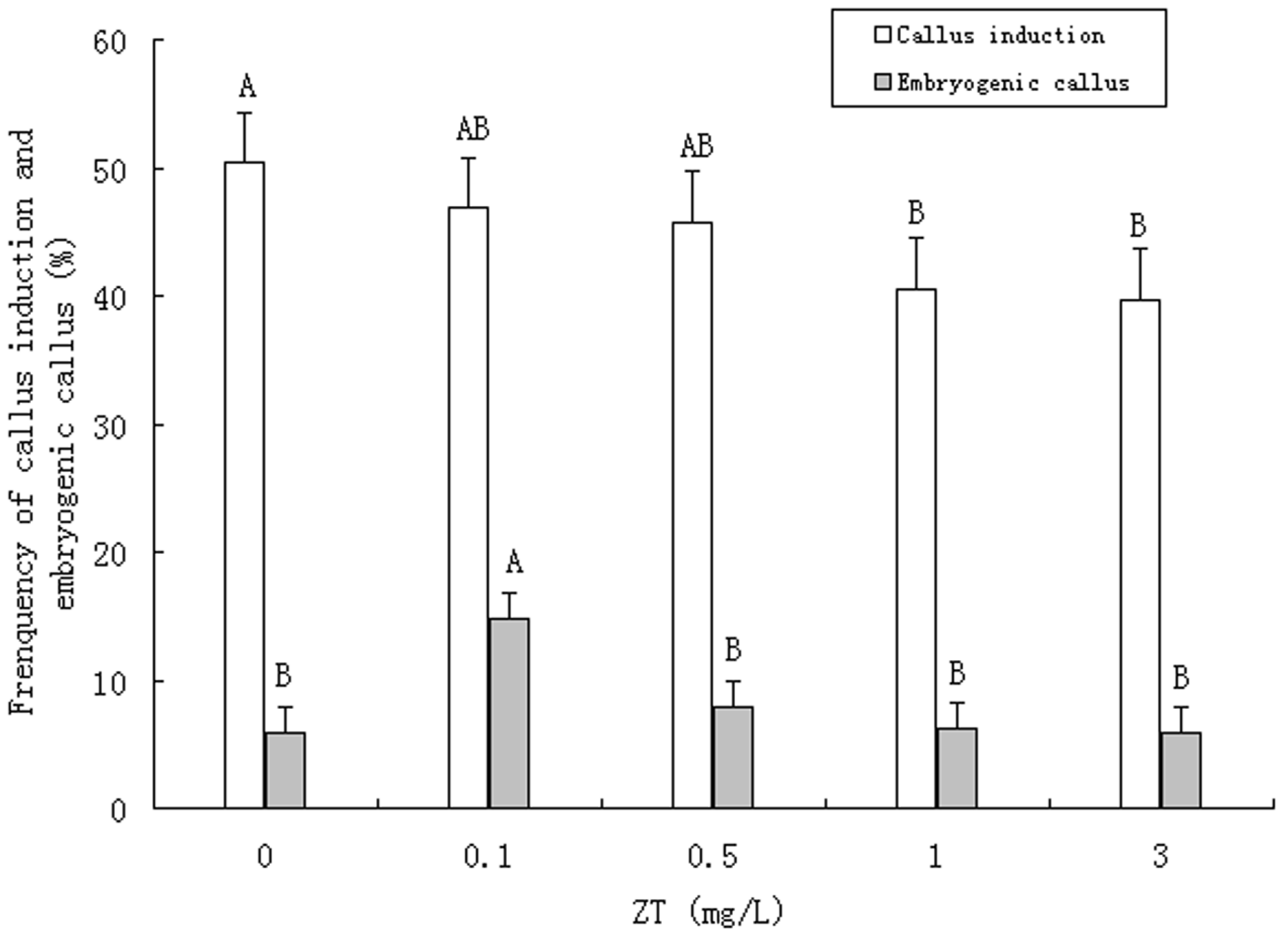
Effect of ZT on callus induction and embryogenic callus formation from zygotic embryos of Moso bamboo*. * Capital letters in the graph mean significant differences at the 0.01 probability level.

Calli formed after 10 d of incubation in medium containing 0.1 mg/L ZT. These induced calli grew faster than the calli derived from the 2, 4-D alone. Effect of ZT on callus induction and embryogenic callus formation were graphed in Figure 3, because the relative number of embryogenic calli decreased as the concentration of ZT increased, the optimal concentration for ZT was set at 0.1 mg/L. In summary, the optimal medium for callus induction of Moso bamboo was MS+4 mg/L 2, 4-D+0.1 mg/L ZT. This media induced embryogenic calli from nearly 15% of explants.

Seeds were used as explants in the present study. Because the juvenile stage for Moso bamboo lasts longer than 60 years and stands die within a year of flowering [29], the availability of starting material for this protocol was limited. However, gregarious or sporadic flowering and seed production of Moso bamboo occurs in China. Moso seeds can be purchased via the Internet or from local seed companies. In the future, further investigations may evaluate the use of vegetative tissues from elite Moso bamboo in order to establish a somatic embryogenesis system for Moso bamboo.

### Callus proliferation and embryogenic calli regeneration

#### Callus proliferation

Embryonic callus cultures of Moso bamboo were grown in Murashige and Skoog media (MS) as described in the materials section with the addition of 2.0 mg/L 2, 4-D. They were subcultured once every 15 days. Continued cultivation for 2 months caused the calli to gradually turn white and show vitrification. In this way, after one month of growth, the 2, 4-D concentration was lowered to 0.5 mg/L for 2 subcultures and then re-adjusted back to 2.0 mg/L. This was repeated, facilitating optimal proliferation of the calli (Figure 1E). During proliferation, darkened calli showing a browning tendency were excluded and vigorous embryonic calli were selected for further subculture.

#### Effect of plant growth regulators on embryogenic calli regeneration

Four kinds of plant growth regulators (KT, BA, TDZ, ZT) were assayed to stimulate callus differentiation. Although calli cultured in the light in the control medium with no hormones for 3 to 5 days became green, and continued cultivation was unable to stimulate proliferative growth, eventually resulting in browning and death. BA (1.0–5.0 mg/L) and KT (1.0–5.0 mg/L) were both ineffective in stimulating callus differentiation and regeneration. About 30% of the calli continued to proliferate for 14 days of incubation with either cytokinin. However, extended cultivation time caused most of the calli to turn brown and die. At high concentrations of BA and KT, calli rapidly became loose and vitrified.

By contrast, TDZ caused the calli to become green: more than 9% of the calli gradually turned green (Figure 1F) after 7 days of cultivation in the TDZ medium at concentrations ranging between 0.05 and 0.1 mg/L. Nearly 27% of the calli greened at 0.5 mg/L TDZ treatments. Greening of callus (%) over the density of TDZ was demonstrated in Figure 2C. However, differentiation was not observed with continuous incubation, and the calli eventually turned brown and died after 20 days in the TDZ treatments.

In medium supplemented with 1.0 mg/L ZT, the calli gradually turned green after 3 to 5 days of incubation. However, there was no shoot regeneration or embryoid formation upon continuous incubation. As the concentration of ZT increased to 2.0–3.0 mg/L, the calli were more likely to turn green, a number of individual calli showed regeneration (Figure 1G) and a few embryoids were induced. When the ZT concentration was 5.0–7.0 mg/L, after 7 to 10 days of cultivation the calli had essentially completed greening. Then only 5% of the calli gradually differentiated into buds directly (Figure 1H), during the differentiation process, most calli gradually turned from transparent or white to yellow-green, light green, or then green. After 20 to 30 days of incubation, approximately 35–37% of the calli had formed embryoids, with each callus mass forming 3 to 12. Effect of ZT on greening of callus, shoot regeneration and embryoid inducement were analyzed in Figure 2D, 2E, and 2F, respectively. Thus, 5.0–7.0 mg/L ZT has been shown to be a positive regulator in Moso bamboo somatic embryogenesis and embryoids germination.

At the initial formation stage of embryoid germination, embryoids were spherical, and individual embryoids tended to separate (Figure 1I and 1J). As cultivation continued, the embryoids gradually developed and elongated, germinating and taking root to become regenerated plantlets (Figure 1K, 1L and 1M). Two types of embryoids were commonly observed in the embryoid formation process: The first type of embryoid was derived from calli gradually forming light yellow, transparent embryoids during cultivation (Figure 1N), which germinated and took root before entering the cotyledon stage. The second type was derived from pale white calli differentiating into white nontransparent embryoids (Figure 1O), which grew rapidly before gradually turning green and germinating. During the development process, the embryoids turned progressively purple at the area connecting the radicle and the germ before elongating further and turning green (Figure 1N and 1O).

#### Effect of carbon source on embryogenic calli regeneration

**Figure 4:**
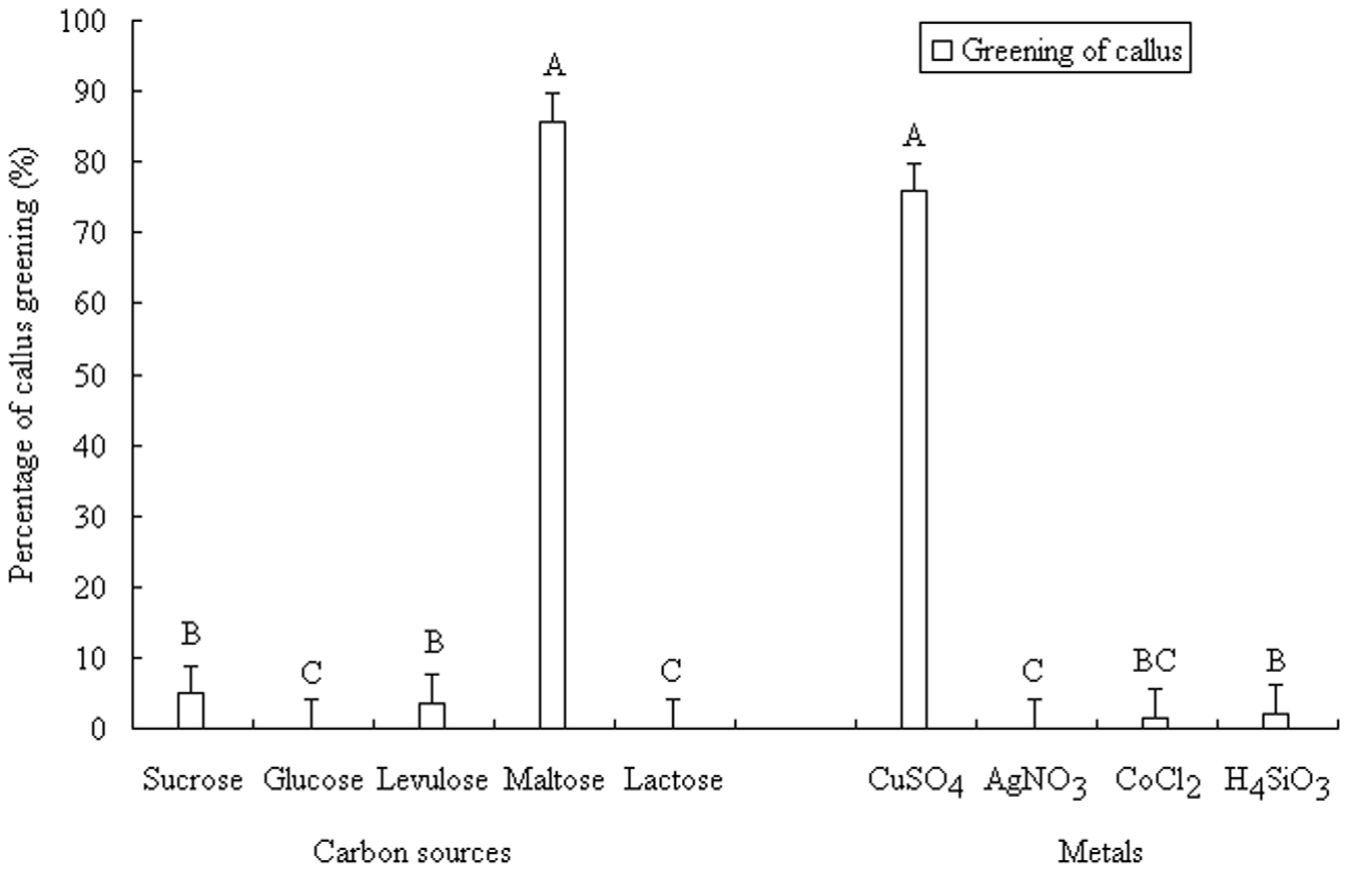
Effect of carbon sources and metals on greening of callus from Moso bamboo*. * Capital letters in the graph mean significant differences at the 0.01 probability level.

A comparison of five carbon sources showed that maltose significantly stimulated callus greening (Figure 4). Green dots on the surfaces of embryoids were observed after 3–5 days of incubation in maltose medium under light cultivation. After 10 days, the entire surfaces of approximately 86% of the calli had become green. As cultivation continued, the green areas of the calli gradually exhibited budding. However, with maltose, the green buds gradually died as cultivation continued. These buds were unable to produce regenerated plants. In the sucrose medium, although there was less greening of the calli than in maltose, the buds were more likely to survive after turning green. By day 20, differentiated buds were perceived, and by day 30, regenerated plants were observed. Vigorous calli growth was observed in media containing fructose, glucose, and lactose, and minimal browning was observed. However, these embryogenic calli exhibited only occasional individual greening, and no differentiation or regeneration was observed. This showed that regenerated plants could only be obtained using sucrose media.

#### Effect of metal on embryogenic calli regeneration

Four trace metals were tested in the culture medium for their effects on calli regeneration (Figure 4). The addition of CuSO_4_ to the sucrose medium significantly stimulated callus greening. Calli cultured in CuSO_4_ began turning green after 5–7 days of incubation, with over 75% greening by 15 d incubation. However, the calli became loose at accelerated growth speeds. No regenerated plants were observed. AgNO_3_ caused most of the calli to turn gray-white and then brown before eventually dying. CoCl_2_ exhibited significant inhibition effects on callus growth, callus greening, or differentiation. When H_4_SiO_3_ was initially added, individual calli turned green. However, browning occurred with continuous incubation. In this way, of the 4 trace additives, only CuSO_4_ showed significant effects on the stimulation of callus greening, but it inhibited further development. H_4_SiO_3_, CoCl_2_, and AgNO_3_ showed negative effects on callus differentiation.

Because the protocol developed herein allows cultivation of Moso bamboo embryonic callus with minimal browning and preserves somatic embryogenesis capability in calli and because its results remained useful for over one year, building a genetic transformation platform through the formation of Moso bamboo embryoids is feasible. Although maltose and copper sulfate exhibit significant effects with respect to stimulating callus greening, they did not induce plant regeneration. These concentrations and length of exposure may require further adjustment. Despite the induction of a large number of embryoids in the MS+5.0–7.0 mg/L ZT media, the embryoid germination rate remained low. Although some embryoids showed normal shoots and roots, most were unable to form regenerated plants and died during the development and germination processes.

## Conclusion

The present report describes the first plantlet regeneration via embryoid germination through callus induction of Moso bamboo, which is one of the most important monopodial bamboos in the world. The seed was used as the explant and incubated in MS medium supplemented with 4 mg/L 2, 4-D and 0.1 mg/L ZT. The callus induction ratio was about 50% and the embryogenic callus induction ratio was nearly 15%. The compact, light yellow embryogenic calli were derived from this medium proliferated in MS medium supplemented with 0.5–2.0 mg/L 2, 4-D. Embryoids formed in MS medium supplemented with 5.0–7.0 mg/L ZT. Nearly 37% of the calli contained embryoids. These embryoids were able to germinate in this medium and developed shoots and roots formation. Just no more than 5.0% of the calli were able to regenerate shoot directly. The regenerated plantlets were subcultured in the MS medium supplemented with 2 mg/L NAA for 15 d to increase the root growth (Figure 1P). These plantlets were capable of being transferred to a greenhouse and surviving acclimatization at a rate of 70% (Figure 1Q). This study is a key first step in the development of Moso bamboo transformation protocols.
